# Clinical and pathological associations with p53 tumour-suppressor gene mutations and expression of p21WAF1/Cip1 in colorectal carcinoma.

**DOI:** 10.1038/bjc.1996.333

**Published:** 1996-07

**Authors:** R. J. Slebos, I. O. Baas, M. Clement, M. Polak, J. W. Mulder, F. M. van den Berg, S. R. Hamilton, G. J. Offerhaus

**Affiliations:** Department of Pathology, University of Amsterdam, The Netherlands.

## Abstract

**Images:**


British Journal of Cancer (1996) 74, 165-171
© 1996 Stockton Press All rights reserved 0007-0920/96 $12.00

### Clinical and pathological associations with p53 tumour-suppressor gene mutations and expression of p21$^{WAF1/Cip1}$ in colorectal carcinoma

RJC Slebos[1], IO Baas[1,2], M Clement[1], M Polak[1], J-W Mulder[1,2], FM van den Berg[1], SR Hamilton[2,3] and GJA Offerhaus[1,2]

*[1]Department of Pathology, Academic Medical Centre, University of Amsterdam, Meibergdreef 9, 1105 AZ Amsterdam, The Netherlands; [2]Department of Pathology and [3]Oncology Centre, The Johns Hopkins University School of Medicine, 632 Ross Research Building, 720 Rutland Avenue, Baltimore, MD 21205, USA.*

**Summary** Inactivation of the p53 tumour-suppressor gene is common in a wide variety of human neoplasms. In the majority of cases, single point mutations in the protein-encoding sequence of p53 lead to positive immunohistochemistry (IHC) for the p53 protein, and are accompanied by loss of the wild-type allele. Recently, the *WAF1/Cip1* gene was identified as one of the genes induced by wild-type p53, and increased expression of p21$^{WAF1/Cip1}$ has been found to reflect the status of the p53 tumour-suppressor pathway. We investigated the inactivation of p53 in a relatively small, but well-characterised, group of 46 colorectal carcinomas that were previously studied for allelic alterations, *ras* oncogene mutations and DNA aneuploidy. Alterations in p53 were identified by IHC, loss of 17p and DNA sequence analysis of exons 5-8, whereas p21$^{WAF1/Cip1}$ protein expression was determined by IHC. p53 mutations were identified in 19 of the 46 tumours (41%), whereas positive IHC for p53 was found in 21 of the 46 tumours (46%). Positive IHC for p21$^{WAF1/Cip1}$ was detected in 16 of 42 cases (38%). We found no relationship between p21$^{WAF1/Cip1}$ staining and p53 protein expression or p53 mutational status. Inactivating mutations in the p53 gene correlated with LOH at 17p but not with LOH at 5q or 18q, Dukes' stage, tumour grade or DNA ploidy. There was a higher survival rate independent of Dukes' stage in the group with no alterations in p53 compared with those with evidence of dysfunction of p53, but the difference was not statistically significant. We conclude that inactivation of p53 and altered expression of p21$^{WAF1/Cip1}$ are common in colorectal carcinoma but do not correlate with each other or with the clinical or pathological parameters investigated.

**Keywords:** p53 tumour suppressor; p21; WAF1; CIP1; colorectal carcinoma

Colorectal cancer is the second most prevalent cancer in the Western world (Boring *et al.*, 1991). Recent studies have elucidated several genetic changes associated with this type of cancer. Activating point mutations in *ras* oncogenes are common in colorectal cancer and are typically found in more than 50% of tumours, whereas loss of heterozygosity (LOH) is frequently found at 5q (APC), 17p (p53), and 18q (DCC) (Vogelstein *et al.*, 1988). According to Knudson's model of suppressor-gene function, such losses are thought to reveal inactivating mutations in the target genes within such regions (Knudson, 1971).

Alterations in the p53 tumour-suppressor gene are among the most frequently encountered genetic aberrations in human malignancies (Hollstein *et al.*, 1991), which suggests a central role for the p53 protein in human carcinogenesis. The wild-type p53 protein suppresses tumour cell growth, binds to specific DNA sequences and participates in cell-cycle regulation (for review see Levine, 1992; Kern, 1994). Inactivating mutations in the p53 tumour-suppressor gene are very frequent in human colorectal carcinomas and are usually accompanied by loss of the remaining wild-type copy of the gene (Baker *et al.*, 1989, 1990; Campo *et al.*, 1991; Cunningham *et al.*, 1992). As early studies indicated that the vast majority of p53 gene mutations are located in exons 5-8, mutational analyses were principally confined to these exons. In addition, not all sequence alterations that lead to an amino acid change in the p53 protein result in impaired p53 function. Examples include temperature-sensitive forms of p53 (Halevy *et al.*, 1990) and mutations that apparently have limited activity in assays for p53 function (Frebourg *et al.*, 1992; Srivastava *et al.*, 1993). For these reasons, mutational analysis of the p53 gene will only reveal a subset of tumours with p53 dysfunction.

A second method for the detection of p53 inactivation is based on the fact that the majority of inactivating mutations in p53 lead to an increased half-life of the p53 protein. Although p53 protein levels in cells with wild-type p53 are too low to detect by routine immunohistochemistry, most p53 gene mutations lead to the accumulation of p53 protein, which is easily detectable with this technique (van den Berg *et al.*, 1989). Because of the relative ease of this approach, a large number of studies have investigated alterations in p53 using immunohistochemistry (Campo *et al.*, 1991; Cunningham *et al.*, 1992; Kaklamanis *et al.*, 1993; Auvinen *et al.*, 1994; Scott *et al.*, 1991; Starzynska *et al.*, 1992; Bell *et al.*, 1993). In some reports these findings were accompanied by a mutational analysis of exons 5-8 of the p53 gene (Cunningham *et al.*, 1992; Cripps *et al.*, 1994). However, the comparison of the results obtained with these studies is often difficult because of the use of different antibodies and experimental conditions that greatly influence the number of p53-positive cells (Baas *et al.*, 1994). It has also been reported that not all inactivating point mutations in p53 lead to protein accumulation detectable by IHC (Rodrigues *et al.*, 1990). In addition, mutations resulting in a truncated p53 protein, such as frame-shift or nonsense mutations, will not yield detectable p53 protein and thus give a false-negative result when only immunohistochemical detection methods are used.

Recently, a gene that can be activated by wild-type p53 was described, *WAF1/Cip1* (El-Deiry *et al.*, 1993; Harper *et al.*, 1993). The *WAF1/Cip1* gene product, p21$^{WAF1/Cip1}$, is a general inhibitor of cyclin-dependent kinases (CDKs), which regulate entry into the DNA synthesis phase of the cell cycle. Apart from induction by wild-type p53, activation of the *WAF1/Cip1* gene can also occur through mechanisms independent of p53 (Dulic *et al.*, 1994; Zhang *et al.*, 1994; El-Deiry *et al.*, 1995). As expression of the *WAF1/Cip1* gene

Correspondence: RJC Slebos, Academic Medical Centre, University of Amsterdam, Department of Pathology, Meibergdreef 9, 1105 AZ Amsterdam, The Netherlands
Received 25 September 1995; revised 25 January 1996; accepted 1 February 1996

is induced by wild-type p53 protein, the levels of p21WAF1/Cip1 protein could potentially reflect the functional status of p53 in cancer cells.

Because of the shortcomings in each assay to test for alterations in p53, we employed all three methods (LOH for 17p, p53 IHC and p53 sequence analysis) to investigate possible alterations in the p53 tumour-suppressor pathway. In addition, we investigated the expression of p21WAF1/Cip1 as a potential marker for the status of p53, a relationship that has not yet been studied in detail. The results from this analysis were compared with alterations in other molecular markers and with clinical and pathological data obtained in previous studies on the same group of colorectal carcinomas.

### Materials and methods

### *Tumour specimens*

A group of 46 colorectal neoplasms collected by the Bowel Tumour Working Group Bank at The Johns Hopkins Hospital (Baltimore, MD, USA) was investigated. The specimens were obtained by surgical resection for adenocarcinoma of the colon or rectum between 1983 and 1987. Previous studies on these samples have been reported for fractional allelic loss (FAL), *ras* point mutations, specific allelic deletions of 5q, 17p and 18q (Kern *et al.*, 1989), DNA ploidy (Offerhaus *et al.*, 1992), and nuclear morphometry (Mulder *et al.*, 1992). FAL was defined as the number of chromosomal arms having allelic loss, divided by the total number of informative chromosomal arms (Kern *et al.*, 1989). There were 24 men and 22 women in the study, between 49 and 83 years of age at the time of surgery. Routine histopathological classification was done on all samples, while staging was according to Dukes (1932).

### *Molecular analysis of the p53 gene*

Somatic mutations in the p53 gene were evaluated in all of the 46 colorectal carcinomas. From routine formalin-fixed paraffin-embedded specimens, areas rich in tumour cells were carefully dissected and used for DNA isolation (Baker *et al.*, 1989; Vogelstein *et al.*, 1988). Exons 5–8 were amplified separately by polymerase chain reaction (PCR) using primers and conditions as described elsewhere (DiGiuseppe *et al.*, 1994). Mutations in the p53 gene were identified by analysing the PCR products by denaturing gradient gel electrophoresis (DGGE) as described previously (DiGiuseppe *et al.*, 1994; Hamelin *et al.*, 1993). Exons positive on DGGE were amplified by PCR using p53-specific primers and cloned into the plasmid vector pBluescript (Stratagene, La Jolla, CA, USA). A minimum of 100 individual bacterial clones were then pooled and used for DNA isolation. Bidirectional DNA sequence analysis was performed using Sequenase Version 2 (United States Biochemicals, Cleveland, OH, USA) (Baker *et al.*, 1990). PCR and sequencing conditions have been described previously (Baas *et al.*, 1995; Kessis *et al.*, 1993).

### *Immunohistochemistry for p53 and p21WAF1/Cip1*

Immunohistochemistry for p53 was performed in all 46 colorectal carcinomas with the antibody DO7 (Dakopatts, Glostrup, Denmark) as described previously (Baas *et al.*, 1994). p21WAF1/Cip1 protein was detected with antibody Ab-1 for WAF1 (Oncogene Science, Cambridge, MA) using citrate buffer for antigen enhancement (Baas *et al.*, 1994), with final detection through standard avidin–biotin staining methods. Slides were scored independently by two observers (GJAO and IOB) and categorised into two groups: no expression (less than 10% positive tumour cells) and positive expression (more than 10% staining tumour cells) (Cunningham *et al.*, 1992; Kaklamanis *et al.*, 1993; Cripps *et al.*, 1994).

### *Statistical analysis*

Differences in frequencies were evaluated by two-sided Fisher's exact test or by $\chi^2$ test for trend. *P*-values less than 0.05 were considered statistically significant. For a multivariate survival analysis, Kaplan–Meier curves were plotted for p53 mutation only, p53 IHC and p21WAF1/Cip1 IHC. In an attempt to compensate for the shortcomings of the assays for p53 status, tumours were also categorised into two groups based upon the number of alteration as measured by the three p53 assays (LOH at 17p, p53 IHC and p53 gene mutation). When two or more of these assays showed alterations in p53, inactivation of the p53 tumour suppressor was considered to be present, whereas the remainder of the tumours were considered to be wild type for p53. A multivariate survival analysis based upon this classification was performed for the total group of tumours, but also for Dukes' B and C cases only, to minimise confounding by stage. Statistical significance of the survival difference was tested by log-rank.

**Table I** Somatic mutations in p53 in colorectal carcinoma specimens

| *Sample* | *Exon* | *Codon* | *Base change* | *Amino acid change* | *p53 IHC*[a] | *Number of 17p alleles* |
|---|---|---|---|---|---|---|
| 4 | 7 | 245 | GGC→GAC | Gly→Asp | 1 | 1 |
| 10 | 7 | 248 | CGG→TGG | Arg→Trp | 1 | 1 |
| 12 | 6 | 189 | GCC→ACC | Ala→Thr | 1 | 1 |
| 15 | 7 | 248 | CGG→TGG | Arg→Trp | 1 | 1 |
| 16 | 5 | 172 | GTT→TTT | Val→Phe | 1 | 1 |
| 18 | 5 | 179 | CAT→TAT | His→Tyr | 1 | 1 |
| 22 | 7 | 248 | CGG→CAG | Arg→Gln | 0 | 1 |
| 26 | 6 | 211 | ACT→GCT | Thr→Ala | 1 | 1 |
| 30 | 7 | 248 | CGG→CAG | Arg→Gln | 1 | 1 |
| 34 | 6 | 198 | GAA→TAA | Glu→STOP | 0 | 2 |
| 35 | 8 | 302 | GGG→G-G | STOP at 344 | 0 | 1 |
| 36 | 6 | 196 | CGA→TGA | Arg→STOP | 0 | 1 |
| 40 | 6 | 216 | GTG→G-G | STOP at 246 | 0 | 1 |
| 41 | 5 | 168 | CAC→TAC | His→Tyr | 1 | 1 |
| | 6 | 191–192 | CTC→- - - | Pro→Del | | |
| 42 | 6 | 220 | TAT→TGT | Tyr→Cys | 0 | 1 |
| 53 | 8 | 286 | GAA→AAA | Glu→Lys | 1 | 1 |
| 58 | 5 | 175 | CGC→CAC | Arg→His | 0 | 1 |
| 61 | 6 | 196 | CGA→TGA | Arg→STOP | 0 | 1 |
| 71 | 8 | 262 | GGT→GAT | Gly→Asp | 0 | 1 |
| | | 269 | AGC→AAC | Ser→Asn | | |

[a]p53 immunohistochemistry negative = 0, p53 IHC positive = 1.

### Results

DNA sequence analysis of p53 revealed missense mutations in 14 samples (30%), while five samples (11%) harboured nonsense mutations or frame shifts resulting in premature protein termination (Table I). In three of these five cases, a simple base change resulted in termination codons (samples 34, 36, and 61), whereas samples 35 and 40 had a one-base pair deletion that altered the open reading frame (Figure 1). For sample number 35 the deletion was in codon 302, leading to an aberrant protein terminating at codon 344, whereas in sample number 40 the deletion was at codon 216, leading to a stop at codon 246. Several samples showed stronger signals for the mutated sequence than for the wild-type sequence on the pooled DNAs, indicating loss of the wild-type allele in these cases (samples 10 and 12). LOH at 17p on DNAs from cryostat sections had been identified previously for these samples (Kern *et al.*, 1989).

Sample number 71 harboured two sequence alterations in exon 8 (Table I). From this sample eight individual plasmid clones were analysed to determine the origin of these two mutations. One of the resulting clones had the wild-type exon 8, while the other seven contained both mutations, indicating that both mutations were originally present in the same p53 allele. Sample number 41 also had multiple mutations: a three-nucleotide deletion in exon 6 that deleted one of two prolines, and two different mutations in exon 5, one in codon 168 (CAC→TAC, histidine to tyrosine) and one in codon 174 that did not result in an amino acid change (AGG→AGA, arginine). Clonal analysis of seven individual plasmid clones showed that the two exon 5 mutations occurred in different DNA strands: the codon 168 mutation was found in one clone, and the codon 174 mutation was found in three clones. The remaining four clones were wild type. Two additional samples harboured gene mutations that did not alter the protein-encoding sequence. Sample number 6 was mutated at codon 217 (GTG→GTA, valine) and sample number 48 had a mutation at codon 197 (GTG→GTA, valine).

To further assess the p53 status of the colorectal carcinomas, the samples were also analysed for p53 protein accumulation using immunohistochemistry. In this analysis 21 samples showed positive nuclear staining for p53 (46%) (Table II). When the presence of p53 gene mutations was compared with positive immunohistochemical staining for the p53 protein, a statistically significant correlation between positive staining and missense mutations was found (Fisher's exact test: $P=0.027$).

When the clinical and pathological parameters were compared with p53 gene mutational status, only one parameter, i.e. tumour size, showed a statistically significant difference between the p53 wild-type and mutant group (Table III). For the comparison of p53 mutations with tumour size, four groups were distinguished: 0–4 cm, 4–6 cm, 6–8 cm and over 8 cm. Mutations in p53 were more often found in smaller than in larger tumours ($P=0.044$, $\chi^2$ test for trend). No differences between the p53-negative and -positive groups were found with respect to Dukes' stage, age at diagnosis, gender or tumour localisation and differentiation.

Mutations in the p53 gene also showed a significant association with loss of the wild-type p53 allele on chromosome 17p (Fisher's exact test: $P=0.016$), whereas no correlation was found between p53 gene mutations and LOH at 5q and 18q (Table IV). Activating point mutations in *ras*

**Table II** Comparison of p53 and p21WAF1/Cip1 immunohistochemistry with p53 gene mutation in colorectal carcinomas

| | *p53 gene mutation* Wild-type | Missense | Nonsense | Total |
|---|---|---|---|---|
| p53 immunohistochemistry[a] | | | | |
| Negative | 16 | 4 | 5 | 25 |
| Positive | 11 | 10 | 0 | 21 |
| Total | 27 | 14 | 5 | 46 |
| p21WAF1/Cip1 immunohistochemistry | | | | |
| Negative | 12 | 11 | 3 | 26 |
| Positive | 12 | 2 | 2 | 16 |
| Total | 24 | 13 | 5 | 42 |

[a]p53 immunohistochemistry compared with p53 wild-type/nonsense and p53 missense mutations: $P=0.027$ (Fisher's exact test).

**Figure 1** Detection of p53 point mutations by DNA sequence analysis of selected DGGE-positive PCR products. (**a**) Example of a point mutation in exon 7 at position 245 (sample 4), and the same region for a sample with a wild-type exon 7 sequence. (**b**) Example of a 1 bp deletion in sample 35 at position 302 leading to a frame shift. The corresponding region of sample 61 indicated the wild-type p53 sequence. The sequence shown was taken from the antisense (reverse) strand.

oncogenes appeared to occur independently of p53 gene mutations, and FAL did not correlate with p53 status (data from Kern *et al.*, 1989). We previously studied DNA ploidy in this group of colorectal tumours (Offerhaus *et al.*, 1992), but when these data were compared with those obtained with the p53 sequence analysis we did not find a correlation between the two parameters.

Survival analysis for p53 gene mutation or p53 IHC did not identify statistically significant differences between the p53-positive and p53-negative groups (data not shown). To compensate for possible shortcomings in these individual assays, two additional groups were composed based upon all alterations found in the p53 locus, in p53 protein expression and by DNA sequence analysis. The 'p53-inactivated' group was composed of 18 tumours with DNA sequence alterations in p53 and allelic loss of 17p, along with eight tumours that had no evidence of p53 sequence mutations but showed a positive p53 IHC and LOH at 17p. Nineteen tumours that lacked p53 sequence mutations and LOH at 17p, and one case (number 34) that harboured a STOP codon but did not show loss of 17p or positive p53 IHC, were classified as 'p53 wild-type'. The Kaplan–Meier curves for survival analysis comparing the two groups are shown in Figure 2a. Although there was a pattern for better survival in patients with an intact p53 tumour-suppressor pathway (the 'p53 wild-type' group), especially after adjustment for Dukes' stage, this did not reach statistical significance (log-rank for the entire group, $P=0.3$; and for Dukes' B and C cases, $P=0.2$). The same trend towards a better prognosis in the p53-negative group was found when only p53 gene mutations were considered.

Forty-two of the 46 samples were available for p21$^{WAF1/Cip1}$ immunohistochemistry, of which 16 (35%) showed positive staining (Figure 3). In all cases positive staining for p21$^{WAF1/Cip1}$ was localised to the nucleus, as was the case for p53 staining. No association between p21$^{WAF1/Cip1}$ protein staining and p53 protein accumulation could be demonstrated in this group of tumours. Thus, mutations in the p53 gene did not preclude increased levels of p21$^{WAF1/Cip1}$ protein expression. In this respect, the five cases with premature termination codons in the p53 coding sequence are of special interest. Two of these stained positively for p21$^{WAF1/Cip1}$ whereas the other three stained negatively, suggesting p53-independent routes of p21$^{WAF1/Cip1}$ induction. Expression of p21$^{WAF1/Cip1}$ did not show a significant correlation with any of

**Table III** Comparison of p53 gene mutations and p21$^{WAF1/Cip1}$ immunohistochemistry with clinical and pathological parameters in 46 colorectal carcinomas

| Parameter | *p53 gene mutation* Wild-type | Mutated | *p21$^{WAF1/Cip1}$ IHC*[a] Negative | Positive |
|---|---|---|---|---|
| Age at diagnosis (median) | 69 | 68 | 69 | 68 |
| Gender (male/female) | 12/15 | 12/7 | 14/12 | 9/7 |
| Size[b] (cm) | 4 | 4 | 4 | 2 |
| Less than 4 | 8 | 11 | 9 | 8 |
| 4–6 | 10 | 3 | 11 | 2 |
| 6–8 | 5 | 1 | 2 | 4 |
| Over 8 | | | | |
| Site (right/left) | 12/15 | 10/9 | 11/15 | 10/6 |
| Differentiation | | | | |
| Good | 2 | 4 | 2 | 3 |
| Moderate | 22 | 13 | 20 | 12 |
| Poor | 3 | 2 | 4 | 1 |
| Dukes' stage | | | | |
| A | 1 | 4 | 4 | 0 |
| B | 10 | 4 | 8 | 5 |
| C | 6 | 5 | 5 | 6 |
| D | 10 | 6 | 9 | 5 |

[a]Data available for 42 cases. [b]p53 gene mutation: $P=0.044$ ($\chi^2$-test for trend).

**Table IV** Comparison of p53 gene mutation and p21$^{WAF/Cip1}$ immunohistochemistry with fractional alleic loss, DNA Ploidy, LOH at 5q, 17p and 18q, and *ras* oncogene mutations in 46 colorectal carcinomas

| Genetic marker | *p53 gene mutation* Wild-type | Mutant | *p21$^{WAF1/Cip1}$IHC*[a] Negative | Positive |
|---|---|---|---|---|
| Fractional allelic loss[b] | | | | |
| < 0.2 | 15 | 9 | 10 | 12 |
| > 0.2 | 12 | 10 | 16 | 4 |
| DNA ploidy[c] | | | | |
| Diploid | 8 | 3 | 4 | 5 |
| Aneuploid | 19 | 16 | 22 | 11 |
| *ras* oncogene mutation[d] | 14 | 5 | 11 | 7 |
| Wild-type | 13 | 14 | 15 | 9 |
| Mutated | | | | |
| LOH (no loss/loss)[b] | | | | |
| 5q | 17/10 | 12/7 | 16/10 | 11/5 |
| 17q[e] | 10/17 | 1/18 | 2/24 | 7/9 |
| 18q[f] | 8/19 | 4/13 | 5/20 | 6/9 |

[a]Data available for 42 cases. [b]Data from Kern *et al.* (1989). [c]Data from Offerhaus *et al.* (1992). [d]Point mutations in codons 12,13 and 61 of the H-, K-, and N-*ras* oncogenes (Kern *et al.*,1989). [e]p53 gene mutation: $P=0.016$ (Fisher's exact test). [f]Data available for 44 cases (p53 mutation), and 40 cases (p21$^{WAF1/Cip1}$IHC).

**Figure 2** Kaplan–Meier survival curve according to the classification of tumours into p53 wild-type and p53-inactivated groups upon assays for LOH at 17p, p53 IHC and p53 sequence mutations of the total group of 46 cases (**a**) (–), p53 negative; (- - -), p53 positive. Survival analysis for 42 cases for which p21$^{WAF1/Cip1}$ IHC was determined (**b**) (–), WAF1 negative; (- - - -), WAF1 positive. Log-rank statistical analysis was not significantly different for both analyses.

a
b

**Figure 3** Immunohistochemistry for p21$^{WAF1/Cip1}$ in colorectal carcinoma and non-neoplastic colonic epithelium. (**a**) the non-neoplastic colonic epithelium shows nuclear staining in the upper crypts and surface where the terminally differentiating cells are located, but epithelial cells in the proliferative zone are negative (original magnification ×140); Panel B: The colonic carcinoma shows nuclear staining of most neoplastic cells but stromal cells are negative (original magnification ×90).

the clinical and pathological parameters (Table III). No difference was observed between the p21$^{WAF1/Cip1}$ IHC-negative and IHC-positive groups when compared with FAL, DNA ploidy and LOH at 5q, 17p, or 18q (Table IV). There was also no difference in survival between the p21$^{WAF1/Cip1}$-negative and -positive group, although the p21$^{WAF1/Cip1}$-positive group appeared to have a slightly better survival than the p21$^{WAF1/Cip1}$-negative group (Figure 2b).

Expression of p21$^{WAF1/Cip1}$ was also found in the crypt epithelium of normal, non-neoplastic colorectal mucosa. Typically, epithelial cells lining the lower two-thirds of the crypts, where proliferation takes place, were negative for p21$^{WAF1/Cip1}$ immunohistochemical staining. Positivity for p21$^{WAF1/Cip1}$ was a consistent finding in the upper one-third of the crypts and the surface epithelium, outside the progenitor zone but where terminally differentiating crypt cells are found. Staining for p53 was always negative in non-neoplastic crypt epithelium.

### Discussion

In this study we investigated the clinical significance of the p53/WAF1 pathway in colorectal cancer. The study group consisted of 46 carcinomas that were previously investigated for loss of heterozygosity at 5q, 17p and 18q (Kern *et al.*, 1989), activating point mutations in *ras* oncogenes (Kern *et al.*, 1989) and DNA ploidy (Offerhaus *et al.*, 1992). Because of these previous studies, this study group provided a unique opportunity to compare alterations in p53 with these previously studied alterations and with the clinical and pathological features in this well-documented group. To our knowledge, this is the first study to compare the clinical relevance of a combination of the three assays for p53 inactivation, in comparison with a spectrum of other genetic alterations.

Abnormalities of the p53 gene can be assessed by DNA sequence analysis, immunohistochemistry to test for p53 protein accumulation and by allelic loss at 17p. Although each of these methods gives an indication of loss of p53 function, they all have limitations. DNA sequence analysis is elaborate and sometimes difficult on archival tumour samples. The mutations found in p53 span several exons, and the presence of contaminating normal cells may dilute the mutant signal. We have employed DGGE to screen for PCR fragments with mutations, a method that reliably identifies exons that are mutated (Hamelin *et al.*, 1993; DiGiuseppe *et al.*, 1994). In our study group, 19 of the 46 colorectal carcinomas (41%) showed inactivating mutations in the p53 gene. This frequency is lower than, but not significantly different from, those reported in the literature (Rodrigues *et al.*, 1990; Lothe *et al.*, 1992; Hamelin *et al.*, 1994). Mutational 'hotspots' for p53 have been defined in previous studies. About one-third of all mutations in colorectal cancer occur in codons 175, 248 or 273. In the present study 5 out of the 19 p53 mutation-positive cases (26%) were mutated in one of these three codons, whereas mutations in other codons that are frequently altered in human neoplasms (codons 179, 196, 220, 245) were also found. The two mutations found in sample number 71 (codons 262 and 269) have not been reported previously. Detailed analysis of these mutations revealed that they were present in the same allele of p53. In contrast, two mutations in case number 41 were present in different alleles. Multiple mutations in one tumour sample have been reported previously (Baker *et al.*, 1990; Hamelin *et al.*, 1994), but their biological significance remains uncertain.

Although DNA sequence analysis of exons 5–8 will detect the majority of the mutations, inactivating mutations in other exons, promoter sequences or positions affecting mRNA splicing may still be present. When such mutations lead to accumulation of p53 protein, detecting by immunohistochemistry will yield more accurate results than DNA sequence analysis. Several immunohistochemical studies have investigated the inactivation of p53. In some of these studies, a positive p53 IHC was associated with shortened survival (Starzynska *et al.*, 1992; Auvinen *et al.*, 1994), although the effect was weak or absent in others (Scott *et al.*, 1991; Bell *et al.*, 1993). We have previously reported a similar study in which we did not find an association between p53 IHC and survival (Mulder *et al.*, 1995). In all these studies, the methods used to detect p53 protein were similar, although different antibodies for p53 were employed. From our own experience, the choice of antibody can greatly influence the number of cases that show positive staining (Baas *et al.*, 1994), and the use of antigen retrieval procedures may lead to an overestimation of the number of p53-positive cases (Baas *et al.*, 1996). LOH at 17p is also associated with p53 mutations (Baker *et al.*, 1990). In one study, p53 gene mutations correlated with shortened survival, 17p allelic loss, location of the tumour and 5q and 18q LOH (Hamelin *et al.*, 1994). Although our study group was limited, a strong association between p53 and 17p LOH was also found, but we did not find any of the other associations. When only p53 sequence mutations were considered, inactivation of p53 appeared to predict a poorer prognosis, although the difference did not reach statistical significance. Of note, in the Dukes' B and C tumours, where a prognostic marker may prove most valuable in clinical management, the differences were not statistically significant, but our study group was small.

One of the main downstream effector molecules of p53 is p21$^{WAF1/Cip1}$, a cyclin-dependent kinase inhibitor. Induction of p21$^{WAF1/Cip1}$ is directly dependent on wild-type p53 protein accumulation, such as occurs after the induction of sublethal DNA damage (El-Deiry *et al.*, 1993). Thus, activation of p21$^{WAF1/Cip1}$ may reflect p53 status of tumour cells. However, when p53 status was compared with p21$^{WAF1/Cip1}$ protein expression no obvious association between the two parameters was found. This finding is in agreement with recently published data, showing that a direct relationship between protein expression of p53 and p21$^{WAF1/Cip1}$ is only observed after sublethal DNA damage, but not under normal conditions (El-Deiry *et al.*, 1995; DiGiuseppe *et al.*, 1995). We observed no association of p21$^{WAF1/Cip1}$ IHC with any of the clinical and pathological parameters, a finding that would limit the use of p21$^{WAF1/Cip1}$ IHC as a specific tumour marker, or as a marker for cancer survival. Recently, p53-independent, cell-type specific pathways leading to the induction of p21$^{WAF1/Cip1}$ have been reported (Michiele *et al.*, 1994; Halevy *et al.*, 1995). Increased levels of p21$^{WAF1/Cip1}$ may not only lead to cell cycle arrest after p53 protein accumulation but may also be linked with terminal differentiation of specialised cells (Parker *et al.*, 1995). Indeed, the highest levels of p21$^{WAF1/Cip1}$ were found in terminally differentiated colonic crypt cells in non-neoplastic mucosa (El-Deiry *et al.*, 1995), as in our study. The observed pattern of p21$^{WAF1/Cip1}$ expression compares well with the mutually exclusive relationship between proliferation and differentiation typical in many different non-neoplastic tissue types, including colonic epithelium.

This study indicates that high p21$^{WAF1/Cip1}$ protein levels are not unique to tumour cells with intact p53 function. In colorectal carcinomas with a mutated p53 gene, cells positive for p21$^{WAF1/Cip1}$ staining probably harbour a functioning differentiation pathway activated through a p53-independent mechanism. Specimens positive for p21$^{WAF1/Cip1}$ showed a heterogeneous staining pattern for p21$^{WAF1/Cip1}$, but the significance of this finding is unclear. The p53 mutational status in colorectal carcinoma cells is not reflected by the levels of p21$^{WAF1/Cip1}$ protein. Our study thus demonstrates that the induction of p21$^{WAF1/Cip1}$ itself through p53-independent mechanisms may occur in a significant fraction of colorectal tumours.

**Acknowledgements**
We thank Dr AC Tersmette and J Oosting for statistical assistance. This work was supported by grant WS92-33 from the Netherlands Digestive Disease Foundation, and grant CA62924 from the National Cancer Institute, National Institutes of Health, USA.

### References

AUVINEN A, ISOLA J, VISAKORPI T, KOIVULA T, VIRTANEN S AND HAKAMA M. (1994). Overexpression of p53 and long-term survival in colon carcinoma. *Br. J. Cancer*, **70**, 293–296.

BAAS IO, MULDER JWR, OFFERHAUS GJA, VOGELSTEIN B AND HAMILTON SR. (1994). An evaluation of six antibodies for immunohistochemistry of mutant p53 gene product in archival colorectal neoplasms. *J. Pathol.*, **172**, 5–12.

BAAS IO, VAN DEN BERG FM, MULDER JWR, CLEMENT MJ, SLEBOS RJC, HAMILTON SR AND OFFERHAUS GJA. (1996). False positive results with antigen enhancement for immunohistochemistry of the p53 gene product in colorectal neoplasms. *J. Pathol.* (in press).

BAKER SJ, FEARON ER, NIGRO JM, HAMILTON SR, PREISINGER AC, JESSUP JM, VANTUINEN P, LEDBETTER DH, BARKER DF AND NAKAMURA Y. (1989). Chromosome 17 deletions and p53 gene mutations in colorectal carcinomas. *Science*, **244**, 217–221.

BAKER SJ, PREISINGER AC, JESSUP JM, PARASKEVA C, MARKOWITZ S, WILLSON JK, HAMILTON SR AND VOGELSTEIN B. (1990). p53 gene mutations occur in combination with 17p allelic deletions as late events in colorectal tumorigenesis. *Cancer Res.*, **50**, 7717–7722.

BELL SM, SCOTT N, CROSS D, SAGAR P, LEWIS FA, BLAIR GE, TAYLOR GRD AND QUIRKE P. (1993). Prognostic value of p53 overexpression and c-Ki-ras gene mutations in colorectal cancer. *Gastroenterology*, **104**, 57–64.

BORING CC, SQUIRES TS AND TONG T. (1991). Cancer Statistics 1991. *Ca*, **41**, 19–36.

CAMPO E, DE LA CALLE-MARTIN O, MIQUEL R, PALACIN A, ROMERO MF, VIVES J, CARDESA A AND YAGUE J. (1991). Loss of heterozygosity of p53 gene and p53 protein expression in human colorectal carcinomas. *Cancer Res.*, **51**, 4436–4442.

CRIPPS KJ, PURDIE CA, CARDER PJ, WHITE S, KOMINE K, BIRD CC AND WYLLIE AH. (1994). A study of stabilisation of p53 protein versus point mutation in colorectal carcinoma. *Oncogene*, **9**, 2739–2743.

CUNNINGHAM J, LUST JA, SCHAID DJ, BREN GD, CARPENTER HA, RIZZA E, KOVACH JS AND THIBODEAU SN. (1992). Expression of p53 and 17p allelic loss in colorectal carcinoma. *Cancer Res.*, **52**, 1974–1980.

DIGIUSEPPE JA, HRUBAN RH, OFFERHAUS GJA, CLEMENT MJ, VAN DEN BERG FM, CAMERON JL AND VAN MANSFELD ADM. (1994). Detection of K-ras mutations in mucinous pancreatic duct hyperplasia from a patient with a family history of pancreatic carcinoma. *Am. J. Pathol.*, **144**, 889–895.

DIGIUSEPPE JA, REDSTON MS, YEO CJ, KERN SE AND HRUBAN RH. (1995). p53-independent expression of the cyclin-dependent kinase inhibitor p21 in pancreatic carcinoma. *Am. J. Pathol.*, **47**, 884–888.

DUKES CE. (1932). The classification of cancer of the rectum. *J. Pathol. Bacteriol.*, **35**, 323–332.

DULIC V, KAUFMANN WK, WILSON SJ, TLSTY TD, LEES E, HARPER JWE AND REED SI. (1994). p53-dependent inhibition of cyclin-dependent kinase activities in human fibroblasts during radiation-induced $G_1$ arrest. *Cell*, **76**, 1013–1023.

EL-DEIRY WS, TOKINO T, VELCULESCU VE, LEVY DB, PARSONS R, TRENT JM, LIN D, MERCER WE, KINZLER KW AND VOGELSTEIN B. (1993). Waf1, a potential mediator of p53 tumor suppression. *Cell*, **75**, 817–825.

EL-DEIRY WS, TAKAHASHI T, WALDMAN T, OLINER JD, VELCULESCU VE, BURRELL M, HILL DE, HEALY E, REES JL, HAMILTON SR, KINZLER KW AND VOGELSTEIN B. (1995). Topological control of p21WAF/CIP1 expression in normal and neoplastic tissues. *Cancer Res.*, **55**, 2910–2919.

FREBOURG T, KASSEL J, LAM KT, GRYKA MA, BARBIER N, ANDERSEN TIB AND FRIEND SH. (1992). Germ-line mutations of the p53 tumour suppressor gene in patients with high risk for cancer inactivate the p53 protein. *Proc. Natl Acad. Sci. USA*, **89**, 6413–6417.

HALEVY O, MICHALOVITZ D AND OREN M. (1990). Different tumor-derived p53 mutants exhibit distinct biological activities. *Science*, **250**, 113–116.

HALEVY O, NOVITCH BG, SPICER DB, SKAPEK SX, RHEE J, HANNON GJ, BEACH D AND LASSAR AB.(1995). Correlation of terminal cell cycle arrest of skeletal muscle with induction of p21 by MyoD. *Science*, **267**, 1018–1021.

HAMELIN R, JEGO N, LAURENT-PUIG P, VIDAUD M AND THOMAS G. (1993). Efficient screening of p53 mutations by denaturing gradient gel electrophoresis in colorectal tumors. *Oncogene*, **8**, 2213–2220.

HAMELIN R, LAURENT-PUIG P, OLSCHWANG S, JEGO N, ASSELAIN B, REMVIKOS Y, GIRODET J, SALMON RJ AND THOMAS G. (1994). Association of p53 mutations with short survival in colorectal cancer. *Gastroenterology*, **106**, 42–48.

HARPER JW, ADAMI GR, WEI N, KEYOMARSI K AND ELLEDGE SJ. (1993). The p21 cdk-interacting protein Cip1 is a potent inhibitor of G1 cyclin-dependent kinases. *Cell*, **75**, 805–816.

HOLLSTEIN M, SIDRANSKY D, VOGELSTEIN B AND HARRIS CC. (1991). p53 mutations in human cancers. *Science*, **253**, 49–53.

KAKLAMANIS L, GATTER KC, MORTENSEN N, BAIGRIE RJ, HERYET A, LANE DP AND HARRIS AL. (1993). p53 expression in colorectal adenomas. *Am. J. Pathol.*, **142,** 87–93.

KERN SE. (1994). p53: tumor suppression through control of the cell cycle. *Gastroenterology*, **106,** 1708–1711.

KERN SE, FEARON ER, TERSMETTE KW, ENTERLINE JP, LEPPERT M, NAKAMURA Y, WHITE R, VOGELSTEIN B AND HAMILTON SR. (1989). Clinical and pathological associations with allelic loss in colorectal carcinoma. *JAMA*, **261,** 3099–3103.

KESSIS TD, SLEBOS RJC, HAN SM, SHAH K, BOSCH X, MUNOZ N, HEDRICK L AND CHO KR. (1993). p53 gene mutations and mdm2 amplification are uncommon in primary carcinomas of the uterine cervix. *Am. J. Pathol.*, **143,** 1398–1405.

KNUDSON A. (1971). Mutation and cancer: Statistical study of retinoblastoma. *Proc. Natl Acad. Sci USA*, **68,** 820–823.

LEVINE AJ. (1992). The p53 tumor-suppressor gene. *New Engl. J. Med.*, **326,** 1350–1352.

LOTHE RA, FOSSLI T, DANIELSEN HE, STENWIG AE, NESLAND JM, GALLIE B AND BORRESEN AL. (1992). Molecular genetic studies of tumor suppressor gene regions on chromosomes 13 and 17 in colorectal tumors. *J. Natl Cancer Inst.*, **84,** 1100–1108.

MICHIELE P, CHEDID M, LIN D, MERCER WE AND GIVOL D. (1994). Induction of WAF1/CIP1 by a p53-independent pathway. *Cancer Res.*, **54,** 3391–3395.

MULDER JWR, OFFERHAUS GJA, DE FEYTER EP, FLOYD JJ, KERN SE, VOGELSTEIN B AND HAMILTON SR. (1992). The relationship of quantitative nuclear morphology to molecular genetic alterations in the adenoma-carcinoma sequence of the large bowel. *Am. J. Pathol.*, **141,** 797–804.

MULDER JWR, BAAS IO, POLAK MM, GOODMAN SN AND OFFERHAUS GJA. (1995). Evaluation of p53 protein expression as a marker for long-term prognosis in colorectal carcinoma. *Br. J. Cancer*, **71,** 1257–1262.

OFFERHAUS GJA, DE FEYTER EP, CORNELISSE CJ, TERSMETTE KW, FLOYD J, KERN SE, VOGELSTEIN B AND HAMILTON SR. (1992). The relationship of DNA aneuploidy to molecular genetic alterations in colorectal carcinoma. *Gastroenterology*, **102,** 1612–1619.

PARKER SB, EICHELE G, ZHANG P, RAWLS A, SANDS AT, BRADLEY A, OLSON EN, HARPER JW AND ELLEDGE SJ. (1995). p53-independent expression of p21-Cip1 in muscle and other terminally differentiating cells. *Science*, **267,** 1024–1027.

RODRIGUES NR, ROWAN A, SMITH ME, KERR IB, BODMER WF, GANNON JV AND LANE DP. (1990). p53 mutations in colorectal cancer. *Proc. Natl Acad. Sci. USA*, **87,** 7555–7559.

SCOTT N, SAGAR P, STEWART J, BLAIR GE, DIXON MF AND QUIRKE P. (1991). p53 in colorectal cancer: clinicopathological correlation and prognostic significance. *Br. J. Cancer*, **63,** 317–319.

SRIVASTAVA S, WANG S, TONG YA, PIROLLO K, CHANG EH, OLSON DC, MARECHAL V, MOMAND J, CHEN J, ROMOCKI C AND LEVINE AJ. (1993). Several mutant p53 proteins detected in cancer-prone families with Li–Fraumeni syndrome exhibit transdominant effects on the biochemical properties of the wild-type p53 identification and characterization of multiple mdm-2 proteins and mdm-2-p53 protein complexes. *Oncogene*, **8,** 2353–2360.

STARZYNSKA T, BROMLEY M, GHOSH A AND STERN PL. (1992). Prognostic significance of p53 overexpression in gastric and colorectal carcinoma. *Br. J. Cancer*, **66,** 558–562.

VAN DEN BERG FM, TIGGES A, SCHIPPER M, DEN HARTOG-JAGER C, KROES W AND WALBOOMERS J. (1989). Expression of the nuclear oncogene p53 in colon tumours. *J. Pathol*, **157,** 193–199.

VOGELSTEIN B, FEARON ER, HAMILTON SR, KERN SE, PREISINGER AC, LEPPERT M, NAKAMURA Y, WHITE R, SMITS AM AND BOS JL. (1988). Genetic alterations during colorectal-tumor development. *New. Engl. J. Med.*, **319,** 525–532.

ZHANG W, GRASSO L, MCCLAIN CD, GAMBEL AM, CHA Y, TRAVALI S, DEISSEROTH AB AND MERCER WE. (1994). p53-independent induction of WAF1/Cip1 in human leukemia cells is correlated with growth arrest accompanying monocyte/macrophage differentiation. *Cancer Res.*, **55,** 668–674.


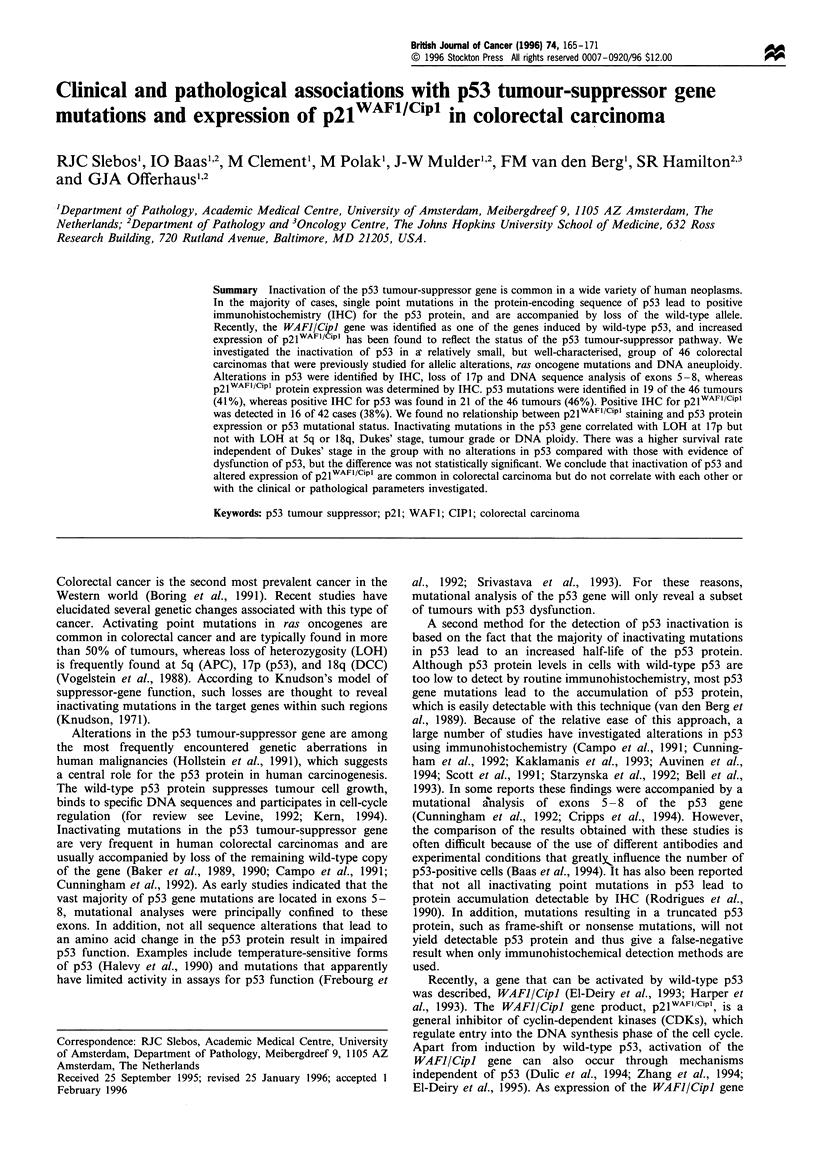

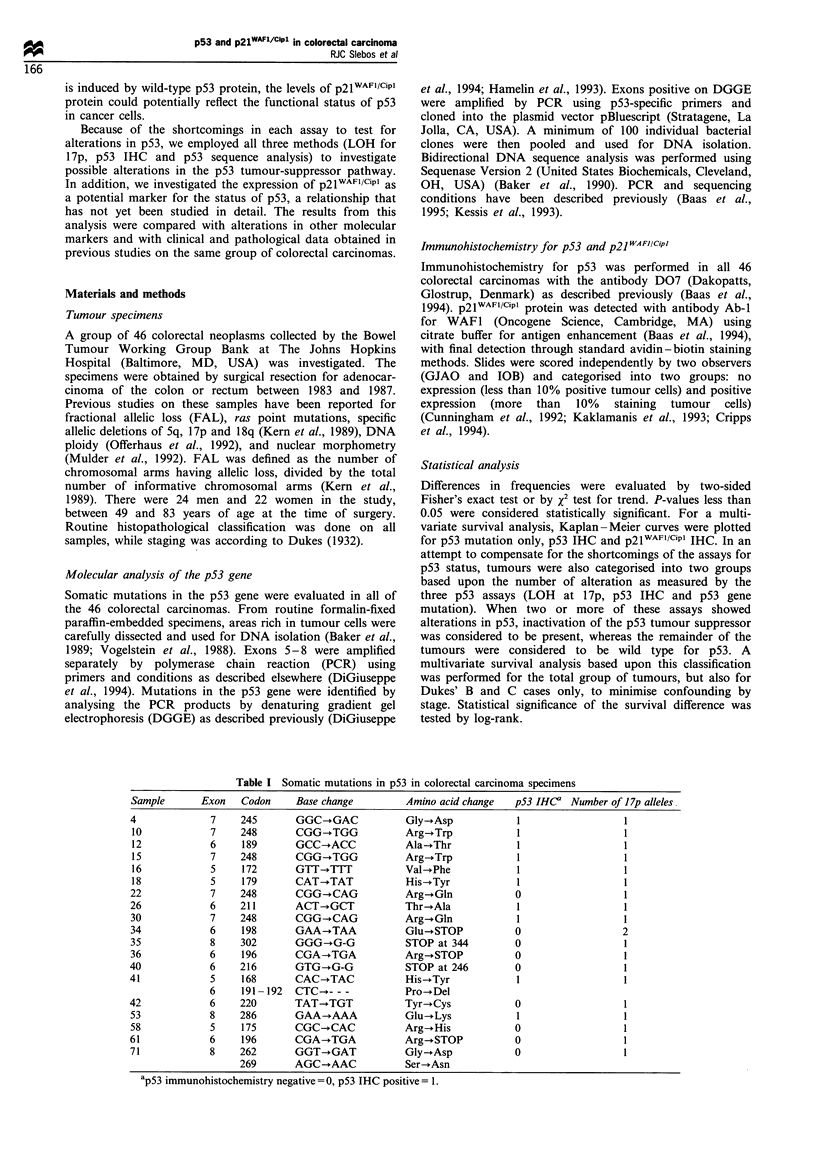

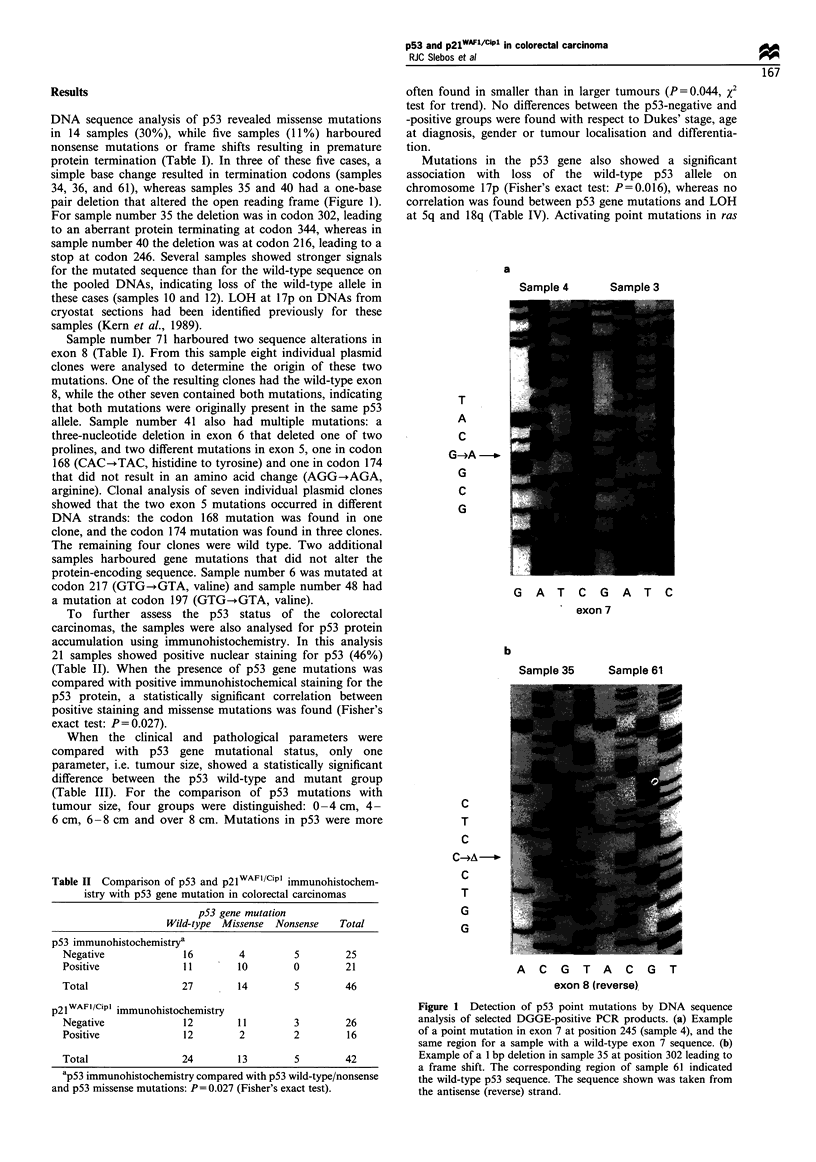

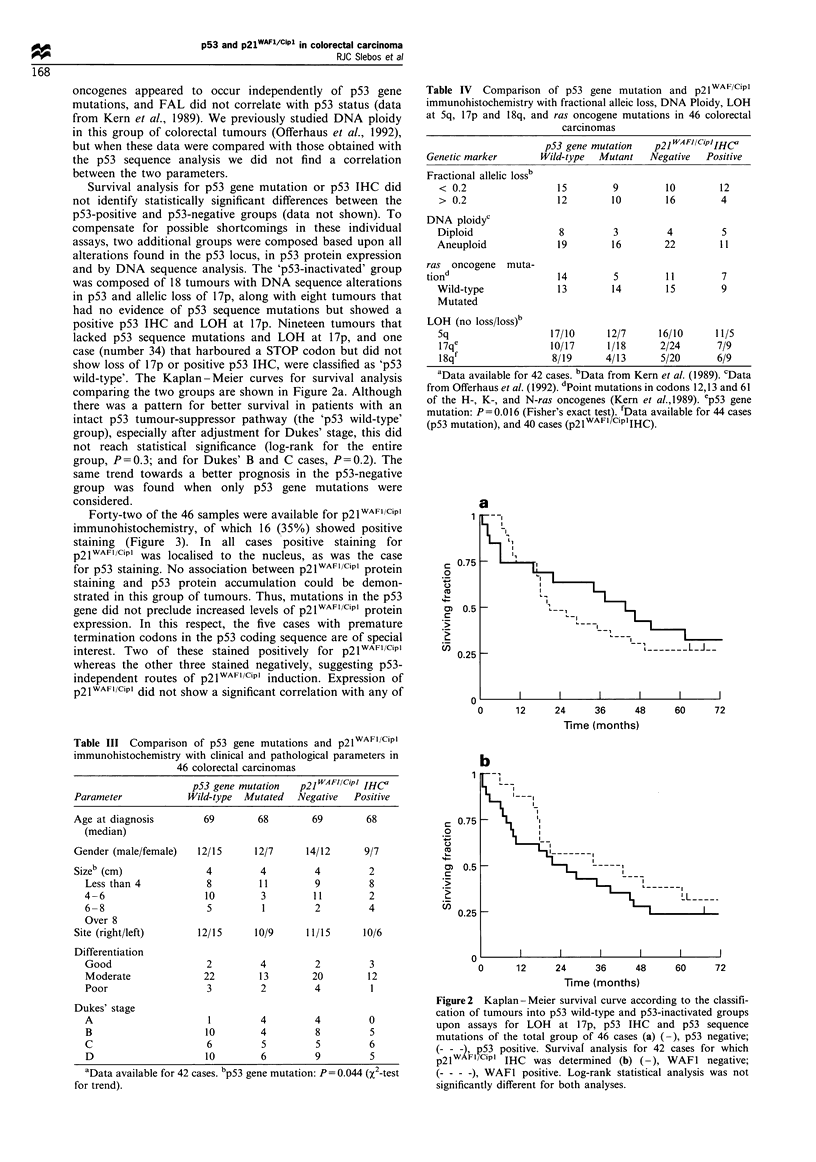

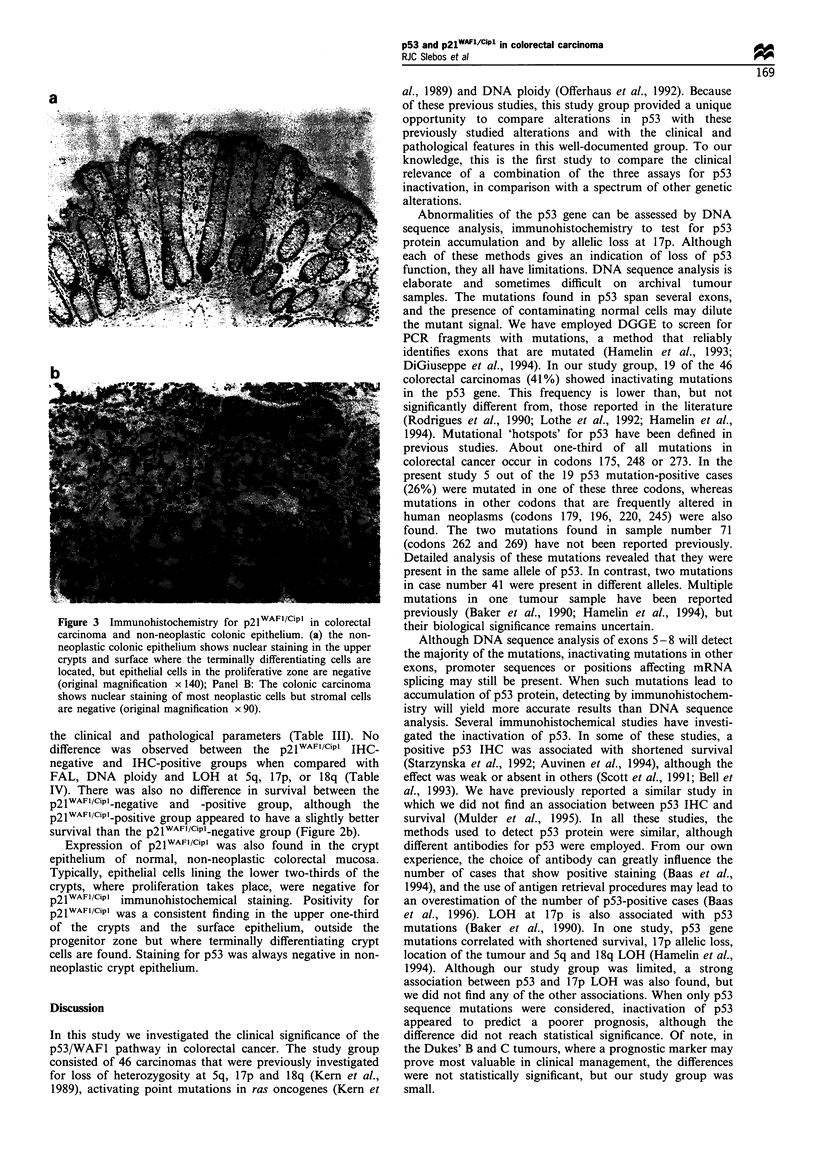

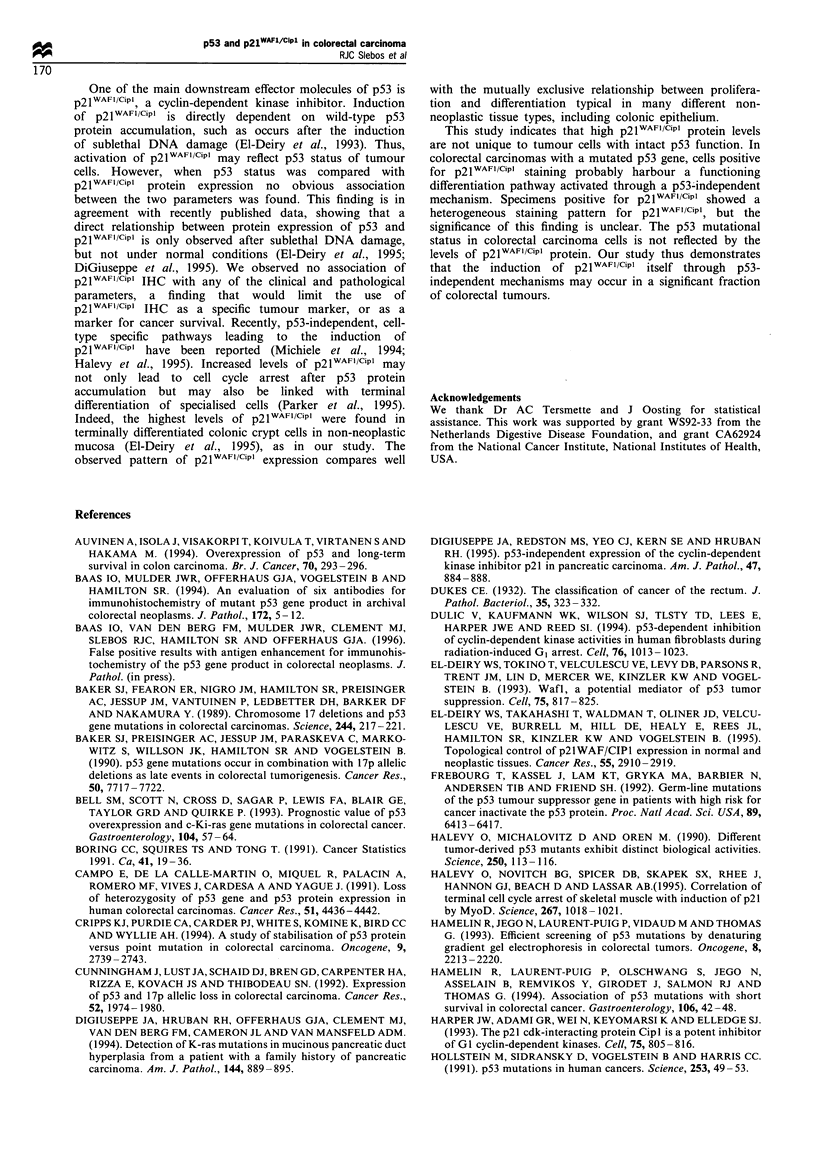

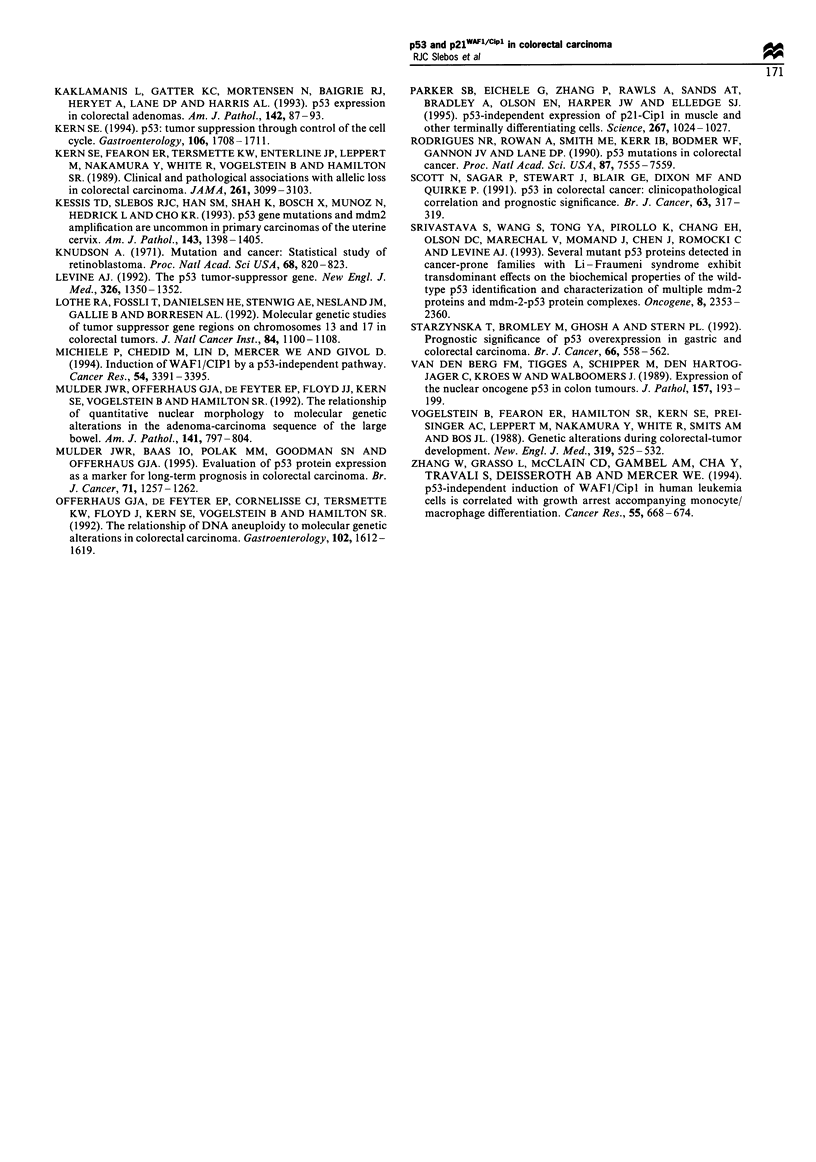


## References

[OCR_00774] Auvinen A., Isola J., Visakorpi T., Koivula T., Virtanen S., Hakama M. (1994). Overexpression of p53 and long-term survival in colon carcinoma.. Br J Cancer.

[OCR_00780] Baas I. O., Mulder J. W., Offerhaus G. J., Vogelstein B., Hamilton S. R. (1994). An evaluation of six antibodies for immunohistochemistry of mutant p53 gene product in archival colorectal neoplasms.. J Pathol.

[OCR_00792] Baker S. J., Fearon E. R., Nigro J. M., Hamilton S. R., Preisinger A. C., Jessup J. M., vanTuinen P., Ledbetter D. H., Barker D. F., Nakamura Y. (1989). Chromosome 17 deletions and p53 gene mutations in colorectal carcinomas.. Science.

[OCR_00798] Baker S. J., Preisinger A. C., Jessup J. M., Paraskeva C., Markowitz S., Willson J. K., Hamilton S., Vogelstein B. (1990). p53 gene mutations occur in combination with 17p allelic deletions as late events in colorectal tumorigenesis.. Cancer Res.

[OCR_00802] Bell S. M., Scott N., Cross D., Sagar P., Lewis F. A., Blair G. E., Taylor G. R., Dixon M. F., Quirke P. (1993). Prognostic value of p53 overexpression and c-Ki-ras gene mutations in colorectal cancer.. Gastroenterology.

[OCR_00808] Boring C. C., Squires T. S., Tong T. (1991). Cancer statistics, 1991.. CA Cancer J Clin.

[OCR_00814] Campo E., de la Calle-Martin O., Miquel R., Palacin A., Romero M., Fabregat V., Vives J., Cardesa A., Yague J. (1991). Loss of heterozygosity of p53 gene and p53 protein expression in human colorectal carcinomas.. Cancer Res.

[OCR_00821] Cripps K. J., Purdie C. A., Carder P. J., White S., Komine K., Bird C. C., Wyllie A. H. (1994). A study of stabilisation of p53 protein versus point mutation in colorectal carcinoma.. Oncogene.

[OCR_00827] Cunningham J., Lust J. A., Schaid D. J., Bren G. D., Carpenter H. A., Rizza E., Kovach J. S., Thibodeau S. N. (1992). Expression of p53 and 17p allelic loss in colorectal carcinoma.. Cancer Res.

[OCR_00833] DiGiuseppe J. A., Hruban R. H., Offerhaus G. J., Clement M. J., van den Berg F. M., Cameron J. L., van Mansfeld A. D. (1994). Detection of K-ras mutations in mucinous pancreatic duct hyperplasia from a patient with a family history of pancreatic carcinoma.. Am J Pathol.

[OCR_00837] DiGiuseppe J. A., Redston M. S., Yeo C. J., Kern S. E., Hruban R. H. (1995). p53-independent expression of the cyclin-dependent kinase inhibitor p21 in pancreatic carcinoma.. Am J Pathol.

[OCR_00850] Dulić V., Kaufmann W. K., Wilson S. J., Tlsty T. D., Lees E., Harper J. W., Elledge S. J., Reed S. I. (1994). p53-dependent inhibition of cyclin-dependent kinase activities in human fibroblasts during radiation-induced G1 arrest.. Cell.

[OCR_00868] Frebourg T., Kassel J., Lam K. T., Gryka M. A., Barbier N., Andersen T. I., Børresen A. L., Friend S. H. (1992). Germ-line mutations of the p53 tumor suppressor gene in patients with high risk for cancer inactivate the p53 protein.. Proc Natl Acad Sci U S A.

[OCR_00875] Halevy O., Michalovitz D., Oren M. (1990). Different tumor-derived p53 mutants exhibit distinct biological activities.. Science.

[OCR_00880] Halevy O., Novitch B. G., Spicer D. B., Skapek S. X., Rhee J., Hannon G. J., Beach D., Lassar A. B. (1995). Correlation of terminal cell cycle arrest of skeletal muscle with induction of p21 by MyoD.. Science.

[OCR_00886] Hamelin R., Jego N., Laurent-Puig P., Vidaud M., Thomas G. (1993). Efficient screening of p53 mutations by denaturing gradient gel electrophoresis in colorectal tumors.. Oncogene.

[OCR_00893] Hamelin R., Laurent-Puig P., Olschwang S., Jego N., Asselain B., Remvikos Y., Girodet J., Salmon R. J., Thomas G. (1994). Association of p53 mutations with short survival in colorectal cancer.. Gastroenterology.

[OCR_00898] Harper J. W., Adami G. R., Wei N., Keyomarsi K., Elledge S. J. (1993). The p21 Cdk-interacting protein Cip1 is a potent inhibitor of G1 cyclin-dependent kinases.. Cell.

[OCR_00903] Hollstein M., Sidransky D., Vogelstein B., Harris C. C. (1991). p53 mutations in human cancers.. Science.

[OCR_00914] Kaklamanis L., Gatter K. C., Mortensen N., Baigrie R. J., Heryet A., Lane D. P., Harris A. L. (1993). p53 expression in colorectal adenomas.. Am J Pathol.

[OCR_00920] Kern S. E., Fearon E. R., Tersmette K. W., Enterline J. P., Leppert M., Nakamura Y., White R., Vogelstein B., Hamilton S. R. (1989). Clinical and pathological associations with allelic loss in colorectal carcinoma [corrected].. JAMA.

[OCR_00916] Kern S. E. (1994). p53: tumor suppression through control of the cell cycle.. Gastroenterology.

[OCR_00926] Kessis T. D., Slebos R. J., Han S. M., Shah K., Bosch X. F., Muñoz N., Hedrick L., Cho K. R. (1993). p53 gene mutations and MDM2 amplification are uncommon in primary carcinomas of the uterine cervix.. Am J Pathol.

[OCR_00932] Knudson A. G. (1971). Mutation and cancer: statistical study of retinoblastoma.. Proc Natl Acad Sci U S A.

[OCR_00938] Levine A. J. (1992). The p53 tumor-suppressor gene.. N Engl J Med.

[OCR_00942] Lothe R. A., Fossli T., Danielsen H. E., Stenwig A. E., Nesland J. M., Gallie B., Børresen A. L. (1992). Molecular genetic studies of tumor suppressor gene regions on chromosomes 13 and 17 in colorectal tumors.. J Natl Cancer Inst.

[OCR_00948] Michieli P., Chedid M., Lin D., Pierce J. H., Mercer W. E., Givol D. (1994). Induction of WAF1/CIP1 by a p53-independent pathway.. Cancer Res.

[OCR_00961] Mulder J. W., Baas I. O., Polak M. M., Goodman S. N., Offerhaus G. J. (1995). Evaluation of p53 protein expression as a marker for long-term prognosis in colorectal carcinoma.. Br J Cancer.

[OCR_00954] Mulder J. W., Offerhaus G. J., de Feyter E. P., Floyd J. J., Kern S. E., Vogelstein B., Hamilton S. R. (1992). The relationship of quantitative nuclear morphology to molecular genetic alterations in the adenoma-carcinoma sequence of the large bowel.. Am J Pathol.

[OCR_00966] Offerhaus G. J., De Feyter E. P., Cornelisse C. J., Tersmette K. W., Floyd J., Kern S. E., Vogelstein B., Hamilton S. R. (1992). The relationship of DNA aneuploidy to molecular genetic alterations in colorectal carcinoma.. Gastroenterology.

[OCR_00974] Parker S. B., Eichele G., Zhang P., Rawls A., Sands A. T., Bradley A., Olson E. N., Harper J. W., Elledge S. J. (1995). p53-independent expression of p21Cip1 in muscle and other terminally differentiating cells.. Science.

[OCR_00980] Rodrigues N. R., Rowan A., Smith M. E., Kerr I. B., Bodmer W. F., Gannon J. V., Lane D. P. (1990). p53 mutations in colorectal cancer.. Proc Natl Acad Sci U S A.

[OCR_00982] Scott N., Sagar P., Stewart J., Blair G. E., Dixon M. F., Quirke P. (1991). p53 in colorectal cancer: clinicopathological correlation and prognostic significance.. Br J Cancer.

[OCR_01000] Starzynska T., Bromley M., Ghosh A., Stern P. L. (1992). Prognostic significance of p53 overexpression in gastric and colorectal carcinoma.. Br J Cancer.

[OCR_01009] Vogelstein B., Fearon E. R., Hamilton S. R., Kern S. E., Preisinger A. C., Leppert M., Nakamura Y., White R., Smits A. M., Bos J. L. (1988). Genetic alterations during colorectal-tumor development.. N Engl J Med.

[OCR_01015] Zhang W., Grasso L., McClain C. D., Gambel A. M., Cha Y., Travali S., Deisseroth A. B., Mercer W. E. (1995). p53-independent induction of WAF1/CIP1 in human leukemia cells is correlated with growth arrest accompanying monocyte/macrophage differentiation.. Cancer Res.

[OCR_00856] el-Deiry W. S., Tokino T., Velculescu V. E., Levy D. B., Parsons R., Trent J. M., Lin D., Mercer W. E., Kinzler K. W., Vogelstein B. (1993). WAF1, a potential mediator of p53 tumor suppression.. Cell.

[OCR_00859] el-Deiry W. S., Tokino T., Waldman T., Oliner J. D., Velculescu V. E., Burrell M., Hill D. E., Healy E., Rees J. L., Hamilton S. R. (1995). Topological control of p21WAF1/CIP1 expression in normal and neoplastic tissues.. Cancer Res.

[OCR_01006] van den Berg F. M., Tigges A. J., Schipper M. E., den Hartog-Jager F. C., Kroes W. G., Walboomers J. M. (1989). Expression of the nuclear oncogene p53 in colon tumours.. J Pathol.

